# Recent Advances in Coronary Chronic Total Occlusions

**DOI:** 10.3390/jcm14051535

**Published:** 2025-02-25

**Authors:** Andreas Synetos, Leonidas Koliastasis, Nikolaos Ktenopoulos, Odysseas Katsaros, Konstantina Vlasopoulou, Maria Drakopoulou, Anastasios Apostolos, Soritios Tsalamandris, George Latsios, Konstantinos Toutouzas, Ioannis Patrikios, Constantinos Tsioufis

**Affiliations:** 1 School of Medicine, European University of Cyprus, 2404 Egkomi, Cyprus; 2 First Department of Cardiology, National and Kapodistrian University of Athens, Hippokration General Hospital of Athens, 11527 Athens, Greece; lkoliastasis@gmail.com (L.K.); nikosktenop@gmail.com (N.K.); odykatsaros@gmail.com (O.K.); vlasopouloukon@gmail.com (K.V.); stsalamandris@hotmail.com (S.T.); glatsios@gmail.com (G.L.);

**Keywords:** chronic total occlusions, CTO, coronary artery disease, percutaneous coronary interventions, complex PCI

## Abstract

Coronary chronic total occlusions (CTOs) have been a point of interest of the medical community for the last decade. The natural history of CTOs was for a long time unknown, as the presence of a single CTO was the most frequent cause for the exclusion of patients from randomized controlled trials (RCTs). Recent CTO RCTs have failed to show any benefit in terms of hard endpoints as major adverse cardiovascular events, but have shown a significant improvement in quality of life, as well in the frequency of angina; however, these studies are characterized by the limitation of the short duration of their follow-up period. Real-world data from observational studies indicate a significant improvement in cardiovascular death and overall mortality, suggesting that the results depend on the duration of the follow-up, and not on the procedure per se. The aim of the current review is to summarize all the existing RCTs, and to analyze the most important registries, as well as to present the current development of techniques to boost the successful interventional treatment of CTOs.

## 1. Introduction

**Figure 1:**
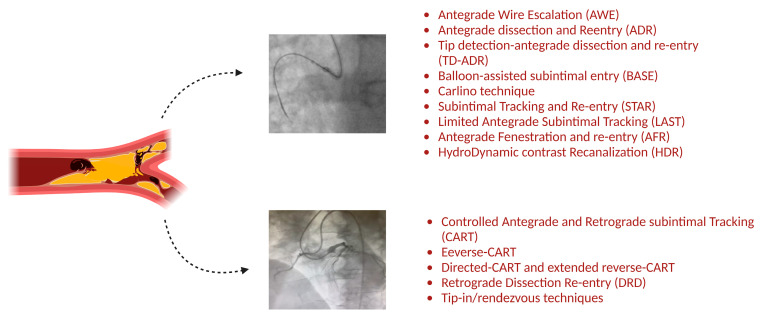
Graphical list of current antegrade and retrograde chronic total occlusion recanalization techniques.

**Figure 2:**
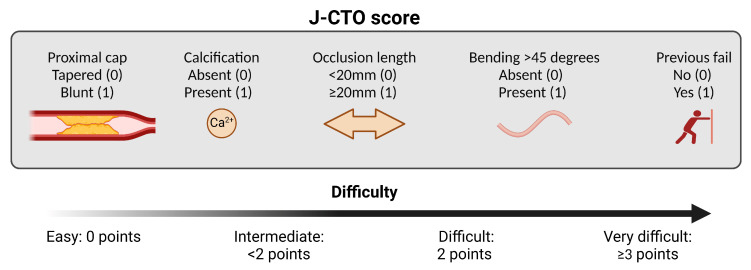
The J-CTO score.

Chronic total occlusions (CTOs), defined as total occlusion of a coronary artery with an estimated or proven presence of at least three months, has a prevalence of 10–35% in patients with coronary artery disease and 54–89% in patients who have undergone coronary artery bypass grafting (CABG) [1,2,3,4]. The evolution of percutaneous coronary interventions (PCIs) for the treatment of CTOs has been a point of interest in interventional cardiology during the last decade (Figure 1). This need was generated due to the fact that although complete revascularization is well known to have better long-term outcomes compared to incomplete revascularization, it is rarely fulfilled after either CABG or PCI procedures [5,6,7,8]. During the beginning of the century, a major drawback that discouraged interventional cardiologists from involvement in CTOs was not only the “early phase” of the materials and the techniques, but mainly the lack of evidence about the natural history of CTOs. In fact, during that time, the presence of at least one CTO was the most frequent reason that patients were excluded from randomized controlled trials (RCTs) [9]. The SYNergy between PCI with TAXUS^TM^ and Cardiac Surgery (SYNTAX) Study, which was designed to determine the best treatment for patients with complex coronary disease by randomizing patients to receive either a PCI with TAXUS stents or CABG, was the first randomized controlled trial that included patients with CTOs, and it was revealed that in the surgical group, only 68.1% of coronary CTOs were revascularized, due to different reasons that were either technically or clinically justified [7,8]. Recent advances in materials, including wires and microcatheters, as well as improvements in the techniques and skills of interventional cardiologists, has resulted in an improvement in the success rate of these complex procedures, with a technical success rate of 85–90%, and a simultaneous improvement in safety, with a complication rate of major adverse cardiac events of 1.5–7% and a mortality rate of 0.2–0.9% [10]. Various CTO scores for predicting the difficulty of recanalization, as well as the possibility of complications, have been developed, such as the J-CTO score and the PROGRESS-CTO score [11,12] (Figure 2). Despite these advances, the wide application of and optimal patient selection for the invasive treatment of CTOs is still questionable, mainly due to a lack of strong evidence that this approach improves patients’ outcomes. In general, the successful revascularization of a CTO improves quality of life and improves symptoms, especially in patients that experience symptoms before the procedure [13,14]. However, even in these settings, it is rather difficult to evaluate the etiology of the symptomatology that patients with a least one CTO experience, as this population tends to underestimate their symptoms because they are well adapted to them [13]. The indications of CTO recanalization were summarized in a recent Consensus document, and are based mostly on symptom relief when the CTO artery supplies a viable segment. In cases of left ventricular dysfunction, significant ischemia and viability, CTO recanalization might be considered in selected cases of young patients with a proximal left anterior descending artery CTO and extended ischemia. However, left ventricular recovery was not confirmed in the randomized REVASC trial, and the concept of myocardial viability remains disputed [15,16,17].

Current guidelines from all cardiology societies suggest that a PCI of a CTO has to be performed only in well-selected patients for the improvement of exercise-limiting symptoms, such as angina. This means that the initial evaluation of the patient requires a careful assessment of their symptoms, as well as of changes in their customary exercise level during recent months. It should be remembered that patients with a CTO often underestimate their symptoms, and often characterize themselves as asymptomatic, since they are used to their symptoms and their clinical status [18]. The use of specific tools, such as the Seattle Angina Questionnaire (SAQ), the EuroQol quality-of-life 5-dimensional score (EQ-5D) or the Rose Dyspnea Scale (RDS), can be helpful for identifying candidates for a PCI for a CTO. Furthermore, screening with a blood test, an ECG, echocardiography or another imaging technique is essential [18,19]. Silent ischemia is a common finding in patients with a CTO, mainly due to adequate collateral circulation during rest, and the finding of a viable myocardium that induces ischemia during stress is essential for the justification of a PCI procedure for a CTO. Moreover, in asymptomatic patients with left ventricular dysfunction, an assessment of ischemia or viability should be performed in all cases, for the identification of patients that will benefit from the procedure [18]. Beyond the presence of ischemia or viability, another parameter that can help to identify the population that will benefit is the electrical stability of the myocardium, as the presence of ventricular arrhythmias have been associated with midterm all-cause mortality [20].

The aim of this narrative review is to analyze the current literature on PCIs for patients with CTOs, examining both RCTs and studies from large-scale registries, and also to provide an analytical approach to the technical aspects of the procedures.

## 2. Randomized Controlled Trials

The establishment of safe and effective PCI procedures for the revascularization of CTOs has opened up a discussion about the necessity of the procedure, as well as about optimal patient selection. Currently, six RCTs (Table 1) have already been conducted that, besides their heterogeneity, agree that the benefit of this procedure is the improvement in quality of life that it provides, mainly due to a decrease in the angina burden, and not improvements in the outcomes of patients.

The DECISION CTO trial was an open-label, multicenter, randomized, non-inferiority trial, in which PCI-eligible patients were assigned to receive either invasive or conservative treatment for their de novo CTO lesion, according to the discretion of the operator. The primary endpoint consisted of the hard composite of death, myocardial infarction, stroke, or any revascularization. The study confirmed the overall safety and efficacy of the procedure, with a success rate of 90.6%, while non-fatal complications associated with the procedure occurred in three patients. However, it failed to show any significant benefit of the CTO PCI strategy compared to the conservative treatment, as after a median follow-up of 4.0 years, there was no significant difference between the two groups (22.3% versus 22.4%, hazard ratio, 1.03; 95% CI, 0.77 to 1.37; *p* = 0.86) [21]. Although the study is generally considered to be a negative trial, this is actually debatable due to several aspects that can be further discussed. The study was prematurely stopped due to enrollment difficulties, with the final number of recruited patients totaling 65% of the estimated sample size. There was a crossover of 15% and 18% between the two arms, but most importantly, the primary endpoints included periprocedural myocardial infarction, which was exclusively responsible for the final differences in non-fatal myocardial infarction. Overall mortality in the PCI arm was lower than that in the conservative arm that received optimal medical treatment (OMT) at both the 4- and 5-year follow-up, while the incidence of stroke was 5-fold higher in the conservative group [21,22]. Therefore, although the study failed to show the non-inferiority of PCIs for CTOs, it pointed out the safety of the procedure, as well as its benefits in terms of reducing mortality at the midterm follow-up.

The EXPLORE (Evaluating Xience and left ventricular function in PCI on occlusiOns afteR STEMI) study tested the improvement of left ventricular function after the successful revascularization of a CTO, by assessing the left ventricular ejection fraction (LVEF) and left ventricular end diastolic volume (LVEDV) by Magnetic Resonance Imaging. The studied population included patients admitted for an ST elevation myocardial infarction that had a CTO in another coronary artery, and their randomization to either a PCI of the CTO, or to the OMT group, was performed after a PCI of the culprit artery. The results were negative in terms of improving primary endpoints after a mean follow-up of 4 months, but a subgroup analysis of the patients who had a CTO in left anterior descending artery showed significant benefits, with improvements in both LVEEV and LVEF (47.2% vs. 40.4%, *p* < 0.05). A serious aspect to take into account when considering the validity of EXPLORE study is the fact that the success rate of the CTOs was relatively low, compared to other randomized trials that were performed around the same time, while the left ventricular remodeling model that was studied during the acute stage of the STEMI was multifactorial, and not ideal for the assessment of the natural history of a CTO. It is also interesting that at the midterm follow-up of the study (3.9 years), patients of the OMT arm had more myocardial infarctions and more coronary artery bypass procedures [23,24]. Besides the overall negative results of the study, the positive results for patients with a CTO located in left anterior descending artery sends a positive message that proper patient selection is essential for increasing survival in this population.

After the relatively negative results of these trials, expectations of organizing a randomized controlled trial with hard endpoints decreased. Therefore, the trials that followed focused on the improvement of quality of life, mainly by decreasing the frequency of angina. The Euro-CTO trial randomized patients to CTO PCI and OMT in a 2:1 randomization, with the primary endpoint being the difference in health status, as assessed by the Seattle angina questionnaire (SAQ) at 12 months. The procedural success was higher than for the EXPLORE trial, reaching 86%, and at the 12-month follow-up, patients in the interventional group had a greater improvement in the SAQ subscale compared to the OMT group. This result was mainly attributed to improvements in their physical limitations and to a decrease in the frequency of angina. The trial did not intend to show any differences in hard events; however, patients in the interventional group had a significantly lower rate of ischemia-driven revascularization compared to the conservative group (2.9% vs. 6.7%, *p* < 0.05) [25].

The REVASC (Recovery of Left Ventricular Function After Stent Implantation in Chronic Total Occlusion of Coronary Arteries) trial evaluated whether a PCI of a CTO, in addition to a PCI of relevant coexisting non-CTO vessels, improves left ventricular function compared to OMT. The primary endpoint of the study was the change in segmental wall thickening (SWT), assessed by MRI. The study had an overall procedural success rate for CTO PCI of 97%, and the authors concluded that there was no benefit from the interventional strategy, as there was no significant difference in SWT between the CTO PCI and no CTO PCI groups (*p* = 0.57). These results are in concordance with other indexes of regional and global left ventricular function, suggesting that there is no additional effect on SWT in the CTO territory. Regarding the clinical effects, and although this was not one of the primary endpoints, the REVASC trial showed a reduction in the combined clinical endpoint favoring CTO PCI, which was driven largely by a reduced need for clinically driven revascularization, with no difference in fatal outcomes [15].

The IMPACTOR-CTO (Impact on Inducible Myocardial Ischemia of PercutAneous Coronary InTervention versus Optimal Medical TheRapy in Patients with Right Coronary Artery Chronic Total Occlusion) trial was a single-center randomized controlled trial that investigated, only for right coronary arteries, the impact on the inducible ischemia burden of PCI versus OMT that included at least two medications with antianginal properties, and the impacts on functional status and quality of life. The primary endpoint of the study was the difference in the myocardial ischemia burden after a follow-up of 1 year, while the secondary endpoints were differences in the six-minute walk test, quality of life and major adverse cardiovascular events. The study was positive in terms of improvement in the myocardial ischemic burden, as at 12 months, there was a significant decrease, from 27.7 ± 8.5% at baseline to 16.1 ± 8.6%, while, similarly, the six-minute walk distance increased significantly in the PCI arm. The results are in concordance with previous studies regarding major adverse cardiovascular events, as no significant difference was found between the two groups at 12 months (94.9% vs. 100%; *p* = 0.19) [26].

**Figure 3:**
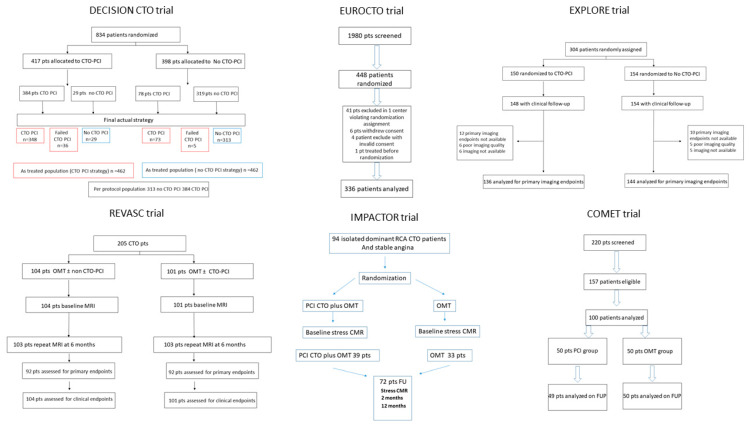
Flowcharts of the randomized controlled trials.

The change in quality of life, assessed using the Seattle Angina Questionnaire, in patients with a successful CTO PCI, compared to patients with OMT, was the point of interest of the COMET-CTO (Randomized Controlled Comparison of Optimal Medical Therapy with Percutaneous Recanalization of Chronic Total Occlusion) trial. This monocentric study had a success rate of 94%, higher than that of previous randomized controlled trials, and resulted in a significant improvement in quality of life in patients who underwent successful PCI of a CTO compared to the control group with OMT. Interestingly, during the follow-up period in the intervention group, no death and low rates of revascularization were recorded. During the follow-up period, the revascularization rates were low for both groups, but they were double in the OMT group compared to the invasive group, with no statistical difference [27]. These results were persistent even for the long-term follow-up of patients, at 56 ± 12 months [28]. Flowcharts of all the RCTs described above are depicted in Figure 3.

It is obvious that after the negative results of the DECISION CTO trial, which was designed to show the non-inferiority of CTO PCIs for hard endpoints, the RCTs that followed focused on lighter endpoints that included parameters of QOL or EF. The drawbacks in the design of the DECISION CTO trial that were mentioned earlier question the global validity of its outcome, and this is partially supported by the results of the other RCTs that have shown benefits for QoL, including a decrease in ischemia and an improvement in EF in specific subgroups.

**Table 1:** Randomized controlled trials for chronic total occlusions. CTO: chronic total occlusion, LVV: left ventricular volume, EF: ejection fraction, MACE: major adverse cardiovascular events, SWT: segmental wall thickness, QoL: quality of life, SAQ: Seattle angina questionnaire, MI: myocardial infarction, LVEF: left ventricular ejection fraction, LVEDV: left ventricular end diastolic volume.

	REVASC [15]	EUROCTO [25]	DECISION-CTO [21]	EXPLORE [24]	IMPACTOR [26]	COMET [27]
Study period	2007–2015	2012–2015	2010–2016	2007–2015	2010–2014	2015–2017
Countries	Germany	Germany, Belgium, Spain, Italy, Greece, France, Denmark	Korea, Taiwan, India, Indonesia, Thailand	The Netherlands, Sweden, Estonia, Norway, Belgium, Canada	Russia	Serbia
Centers	Single center	Multicenter	Multicenter	Multicenter	Single Center	Single
No.	205	396	815	150	317	100
ACS patients	No	No	Yes	Yes	No	No
Outcome	Primary: change in SWT in the CTO territory. Assessed by cMRI. Secondary: improvement in regional wall motion and changes in LVV and EF.	Primary: change in QoL assessed by SAQ. Secondary: MACE	Primary: composite of death from nay cause, MI, stroke or revascularization. Secondary: bleeding, stent thrombosis and QoL.	Primary: LVEF and LVEV assessed by MRI.	Primary: decrease in NIB after CTO PCI. Secondary: changes in 6 min walk test, MACE and QoL.	Primary: QoL. Secondary: all-cause death, MI and recurrent revascularization.
Follow-up	6 months for primary endpoints, 12 months for MACE	12 months	4 years	4 months	12 months	275 days
Results	No differences in primary endpoints and LVV. Significantly lower MACE in PCI group at 12 months.	Greater improvement in SAQ observed with PCI as compared with OMT. No differences in MACE.	No differences in primary or secondary outcomes.	No differences in LVEF and LVEDV. Subgroup analysis showed increased LVEF and LVEDV when CTO was in LAD.	Significant decrease in MIB. Significant improvement in 6 min walk test and QoL. No difference in MACE-free survival.	Improvement in QoL in patients with CTO PVI. No differences in secondary endpoints.

## 3. Real-World Data

The potential benefits of the successful revascularization of a CTO have been the point of interest of many retrospective studies and registries, with the majority of them suggesting that this procedure may be associated with improved clinical outcomes, including a decrease in the frequency of angina and improvements in exercise capacity and in the function of the left ventricle, as well as decrease in the ischemic burden and electrical instability [29,30]. It is of great interest that the longer the follow-up of patients, the greater the likelihood of a positive effect, even on mortality [31]. The main limitation of much of the retrospective data is that some of the studies, especially in the early era of CTO studies, had a selection bias, as they compared patients with successful versus failed CTO PCIs.

The OPEN-CTO (Outcomes, Patent Health Status, and Efficiency in Chronic Total Occlusion Hybrid Procedures) tested the feasibility and outcomes in terms of quality of life as reported by 1000 consecutive patients in expert US centers. This registry had a success rate of 86% and a relatively high percentage of complications that reached 7%. One month from the randomization, CTO PCI improved patients’ health status, as assessed by the SAQ. A successful PCI of a CTO resulted in an increase in the SAQ score compared with an unsuccessful PCI, a difference that is considered clinically meaningful, while dyspnea, which is present in up to 50% of CTO patients, improved in 70% of cases and disappeared in over 42%. The psychological burden had an important effect on the frequency of experiencing angina. Patients that were diagnosed with depression at baseline experienced angina more frequently, and, during follow-up, showed a greater improvement in symptoms compared to non-depressed patients [32,33].

The PROGRESS-CTO (Prospective Global Registry for the Study of Chronic Total Occlusion Intervention) tested the clinical and angiographic characteristics and procedural outcomes in 12,000 patients between 2016 and 2021, mainly in the USA, but also in other countries. The patients included in the registry had a high prevalence of coronary intervention, either PCI (62%) or previous coronary artery bypass graft surgery (29%). The technical success rate was 87%, interestingly increasing from 81.6% in 2016 to 88.1% in 2021, with lower in-hospital major cardiovascular adverse events compared to the OPEN-CTO registry (2.1%). In-hospital mortality was 0.4%, and was correlated with history of heart failure (43% versus 28%; *p* = 0.023). During an average follow-up period of 3.4 years the incidence of major adverse cardiovascular events (MACEs) at 1, 2 and 3 years was 12%, 23% and 27%, respectively, and these were noticed mainly in patients with more complex lesions [11,34].

The question of improving survival following vascularization of a CTO is still unanswered, as the relatively short duration of follow-up might influence the neutral effect of CTO recanalization on mortality [35,36]. In-hospital mortality, as well as long-term major adverse cardiac events and all-cause mortality, were assessed in an observational study that included 5500 PCIs of CTOs, performed in United Kingdom between 2005 and 2015. A success rate of 74.3% was achieved, and after a median follow-up of 4.8 years, successful CTO PCIs were associated with a 9.5% lower mortality rate that persisted after multivariate Cox analysis [37]. In the same region, a similar observational study conducted approximately in the same era concluded that after a follow-up of 4 years, CTO recanalization not only significantly improved quality of life, in terms of physical activity limitation, frequency of angina and greater treatment satisfaction, but also showed a trend towards better cardiac survival and significant lower risk of MACE compared to that seen in patients with failed procedures [38]. Moreover, in an analysis of the U.K. Central Cardiac Audit Database for all CTO PCI cases carried out in England and Wales between 1 January 2005 and 31 December 2009 (13,443 patients), during a follow-up period of 2.65 years, a successful PCI of at least one CTO was associated with improved survival, compared to patients that had a failed PCI for a CTO (hazard ratio [HR]: 0.72; 95% CI: 0.62 to 0.83; *p* < 0.001) [39]. These results are in concordance with a multicenter registry performed in New York, South Korea and Italy, where a 5-year follow-up of 1226 patients showed that successful CTO PCIs were associated with lower cardiac mortality (hazard ratio [HR]: 0.40 versus 0.75, *p* = 0.01) and a reduced need for coronary artery bypass graft surgery (HR: 0.21, versus 0.40, *p* = 0.01) [40]. The IRCTO (Italian Registry of Chronic Total Occlusions) prospectively enrolled 1777 patients with a least one CTO, and at the short-term follow-up, the results showed that major adverse cardiac and cerebrovascular events were significantly less common in the PCI group compared to the OMT group and to the group that underwent coronary artery bypass grafting (2.6% vs. 8.2% and vs. 6.9%; *p* < 0.001 and *p* < 0.01). Patients in the PCI group had significantly lower percentages of cardiac death and myocardial infarction, as well as lower rates of hospital readmission [41].

One of the largest observational studies on CTO PCIs, which was focused on long-term outcomes (>5 years) following CTO PCIs, was performed by Park et al. This study included 1547 patients, with a mean follow-up of 7.9 years, and compared PCI in a CTO artery to OMT. The success rate was 79%, and the rate of cardiac death, which was defined as a primary endpoint of the study, was significantly lower in the PCI group compared to the control group (10.4% versus 22.3%, *p* < 0.001). The study revealed a fact that is obvious in the cardiological community, which is that the longer the follow-up period, the greater the benefit from the revascularization of a CTO. Indeed, a difference in cardiac mortality was noticed between the third and the tenth year, while during the first 3 years, no benefit of a CTO PCI was observed (4% versus 6.5%; *p* = 0.61) [42]. The main differences with the RCTs was that the patients of the registry were younger, with lower comorbidities, higher ejection fractions and lower percentages of known coronary artery disease and previous PCIs; these patients represent a population that seems to have greater long-term benefits, as they have a higher life expectancy compared to people with older age and more comorbidities [42,43]. The impact of the duration of the follow-up, in randomized controlled studies, on cardiac mortality in patients with chronic coronary syndromes undergoing PCIs compared to OMT, has been studied in a recent meta-analysis, which concluded that for each 4 years of follow-up, there was a decrease in cardiac death by 19% in favor of the revascularization group [35]. This is also supported by the results of the long-term follow-up of the ISCHEMIA-EXTEND trial, where patients showed a 12% lower 7-year rate of cardiovascular mortality with an initial invasive strategy compared to OMT [44]. Recently, a study by Yang et al. confirmed that the long-term benefits of revascularization for CTO are evident in patients with multivessel disease, as it reduces the incidence of all-cause death, myocardial infarction and a composite of both over a 5-year follow-up, compared to medical treatment for CTO lesions [45].

## 4. Antegrade Techniques

The antegrade CTO technique—previously referred to as the antegrade wire escalation technique—is the most commonly used approach to CTO recanalization [46,47]. Contemporary expert consensus documents provide structured algorithms to guide operators through selection from various CTO techniques [48]. The parameters to be taken into account in favor of an antegrade initial approach are a lack of ambiguity of the proximal cap, good distal vessel quality, a lack of major side branches at the distal cap, a lack of good interventional collaterals and a <20 mm length of the occlusion [47,49]. The use of dual injections is based on collaterals that provide a view of the distal vessel, except for cases with ipsilateral collaterals. Moreover, the terms “intimal/subintimal” have been substituted by the more suitable terms “intraplaque/extraplaque” to define the route of the wire or materials at the site of the occlusion.

Short and well-defined CTOs are more likely to be penetrated by the simplest version of antegrade techniques, antegrade wiring. This is suggested as the first step in CTO recanalization, particularly for operators at the beginning of their learning curve [50]. With the support of a microcatheter, initially, a workhorse wire is delivered just to the proximal cap, and then exchanged for a dedicated CTO wire. A key factor in this technique is the strong support that can be achieved by a proper microcatheter, a larger guide catheter, femoral access, guide extensions and techniques such as anchoring and dedicated microcatheter manipulation through the plaque. The choice and escalation of the wire is based on the CTO characteristics, the operators’ familiarity and the cath lab’s material availability. A dynamic approach with interchange among different wires is necessary, and a predefined maximum duration of persistence should be followed for each one [51]. This is a general rule for CTO interventions; operators should not stick to a technique that fails for a long time during the procedure. The predominant wire types are polymer-jacketed wires (tapered and non-tapered with different tip loads) and tapered non-polymer wires with high torqueability and higher tip load (Table 2 and Table 3) [52]. Polymer-jacketed wires can be navigated through loose tissue and are less likely to exit the vessel architecture, but may bounce off at a resistant proximal cap. On the contrary, non-polymer-jacketed tapered wires with increased tip load may penetrate a fibrocalcific proximal cap, but an increased penetration force is accompanied by an increased risk of exiting the vessel architecture [53]. Knowledge of microcatheter technologies increases success rates, especially in cases of an ambiguous proximal cap, resistant and angulated lesions and the presence of side branches (Table 4) [54,55]. Dual lumen that are angulated, steerable and of different sizes, and support microcatheters, utilized based on the plaque characteristics, are critical assets in each antegrade or retrograde technique. The proximal cap puncture may also be assisted by the use of Computed Tomography Coronary Angiography (CTCA) and Intravascular ultrasound (IVUS). CTCA can define the proximal cap characteristics, the presence of side branches and tortuosity, and is associated with increased procedural success rates and reduced complications [56,57]. IVUS-guided proximal cap puncture is useful in stumpless CTOs and in cases of deflection of the wire in side branches, such as septals and marginals. The puncture may be performed under direct IVUS imaging, but a catheter larger than 6F is required to accommodate an IVUS catheter and a microcatheter [58,59].

Another approach to antegrade CTO is extraplaque navigation, which is based on creating a dissection plane. The parallel wiring technique is the oldest technique, and is a precursor of antegrade dissection strategies. It is not infrequent, also, for an antegrade wiring approach to end up in a dissection plane, and then regain an intraplaque track, or re-enter the true lumen of the vessel beyond the occlusion. In the parallel wiring technique, the wire that has inadvertently created a dissection stays in the extraplaque space (works as a reference wire) and, with another, using a microcatheter, is advanced to penetrate the proximal cap. Contemporary modification uses a dual-lumen microcatheter to direct the second wire to the intraplaque space. For this purpose, the second wire is tapered, with a higher tip load, to penetrate the plaque. In the Progress CTO registry, parallel wiring was used in 40% of the patients, whereas the Antegrade Dissection and Re-entry (ADR) technique was performed in the rest. ADR exhibited higher procedural success rates, but more complications [60]. In the latter, the wire navigates the extraplaque space and re-enters distally in a “true-to-true” fashion. To create a dissection intentionally, a low-lip-load, polymer-jacketed wire is pushed and knuckled within the occlusion, or, rarely, a dedicated device (CrossBoss, Boston Scientific) is used [61]. Other ways to enter the extraplaque space are so-called “move the cap” techniques [62]. With the scratch-and-go technique, a high-tip-load wire enters directly the extraplaque space. In the balloon-assisted subintimal entry (BASE) technique, a slightly oversized balloon is dilated proximally to the occlusion to create diffuse microdissections, which can be entered with polymer-jacketed guidewires [63]. The Carlino technique also used for uncrossable lesions, which uses forced contrast injection to modify the plaque [64]. A modification–evolution of the Carlino technique is the HydroDynamic contrast Recanalization (HDR) technique, in which a microcatheter is advanced into the CTO body, and a gentle contrast injection creates microchannels, enabling polymer-jacketed wires to cross [65]. The controlled use of ADR mandates the use of the dedicated re-entry Stingray device (Boston Scientific) and a high-tip-load guidewire. The Stingray balloon is placed in the subintimal space distal to the occlusion, and a high-tip-load wire is used to penetrate the intima and establish access to the true lumen [66]; this wire is associated with fewer adverse events, but the use of this device demands a significant learning curve, and it is not available in every cath lab [67]. Alternative strategies involving the use of a dual-lumen microcatheter or the ReCross device (IMDS) have been introduced with, very good results [68]. The RESHAPE registry, however, indicates that ADR should not be considered as a sole method, but as a significant parameter of a holistic hybrid CTO approach [69]. The way in which re-entry is achieved defines different aspects of dissection strategies. The Subintimal Tracking and Re-entry (STAR) technique was introduced by Colombo in 2003, and describes navigating in the extraplaque space with a knuckled wire that re-enters the distal true lumen. Advances and modifications of the STAR technique are mini-STAR and the contrast-guided STAR, whereas in the LAST technique, re-entry is achieved with high-tip-load wires with a bended tip, and this technique has been abandoned due to its unpredictability [64,70,71,72]. IVUS may facilitate re-entry by allowing penetration of the distal lumen under direct imaging; however, this technique requires high experience and skill, and is limited to high-volume centers. Using the tip detection–Antegrade Dissection and Re-entry (TD-ADR) technique allows visualization in real time of the antegrade guidewire through the IVUS, ensuring that is can be guided accurately. The IVUS catheter is placed either in a side branch or in the subintimal space [73]. Finally, the Antegrade Fenestration and Re-entry (AFR) technique is based on inflating a 1:1 (sized with the arterial diameter) balloon into the extraplaque space to create multiple fenestrations, and advancing a soft polymer-jacketed wire directly to the true lumen through them [74]. AFR has been found to be moderately effective (65.9% of cases were successful) and safe, and does not preclude the use of other CTO techniques [75]. Despite the fact that extraplaque techniques increase the rates of antegrade CTO recanalization, they are more complex and are associated with higher complication and target lesion revascularization rates [76].

## 5. Retrograde Techniques

Retrograde strategies have evolved to become an essential tool for CTO recanalization, and have improved the success of CTO interventions to 85–90% in experienced centers [77,78]. Algorithms of CTO crossing have been developed and published by multiple CTO clubs, and there is a general consensus that a retrograde approach should be selected for long lesions with appropriate collaterals and ambiguous proximal caps, as well as in cases of failed antegrade attempts [48]. The selection of the collateral route is of utmost importance, and instrumenting collaterals to cross the occluded lesion requires crossing materials through the donor vessel, thus potentially compromising its integrity [79]. Septal connections are preferable, because of their more controllable complications; however, epicardial vessels may be crossed if suitable. Major factors that affect the selection include the connection size, the tortuosity and the operator’s experience [80,81]. A microcatheter should reach the occluded artery at the level of the distal cap, and thin tapered microcatheters have improved success rates. Forcing a microcatheter through thin and tortuous collaterals should not be attempted, as it may lead to damage by perforating and overstretching [51].

The initial Controlled Antegrade and Retrograde subintimal Tracking (CART) technique required dilatation of the septal collateral, whereas the development of wire and microcatheter technology has led to less traumatic contemporary techniques [63]. CART is based on delivering an antegrade and retrograde wire into the occluded segment, and is facilitated by balloon inflation via the retrograde wire into the occluded segment (intraplaque or extraplaque) to achieve the “meeting” of the two wires. The modern reverse-CART technique involves the balloon being delivered and inflated antegradely, to allow the retrograde wire to find its passage into the proximal vessel [82]. Directed CART and extended reverse-CART are different aspects of the CART technique, and a recent consensus document attempted to define the terminology [83]. IVUS may also facilitate the identification of the two wires at the “meeting point” [84].

The Retrograde Dissection Re-entry (DRD) technique is very similar, and is often confused with the CART technique. In DRD, two knuckled antegrade and retrograde wires are delivered to the occluded segment, reducing time and materials. Care must be taken to avoid subintimal tracking in places with major side branches. Sometimes, it is the only feasible retrograde technique, such as in cases of long, ambiguous courses and calcified lesions [46,85].

The final step of the CTO retrograde technique is the externalization of the retrograde wire. This can be achieved using tip-in/rendezvous techniques. In the tip-in technique, the retrograde wire is inserted into the antegrade microcatheter, which feeds into the antegrade guide catheter. In the rendezvous technique, the antegrade wire is introduced into the retrograde microcatheter. Ultimately, the system is converted into an antegrade approach [86]. Nevertheless, in cases of ostial lesions or unstable catheter intubation, the retrograde wire can be advanced to the ascending aorta and snared through the antegrade catheter [87]. The dedicated RG3 wire (Asahi Itecc) is the preferred wire for externalization, but in the published literature, other wires such as Rotawire can also be used with great caution, and ensuring that these wires are always protected by a microcatheter during collateral surfing [88].

Compared to antegrade techniques, retrograde techniques are associated with higher risk and long-term adverse events [89]. Specific CTO characteristics, though, mandate the use of the retrograde approach, which should be performed by experienced operators, as it reached rates of 5.8% for perforation and 3.5% for periprocedural adverse events in a large recent registry [90,91]. The Global CTO Algorithm was presented by Wu et al. in an effort to create a more standardized approach to CTO recanalization using the available techniques [48]. It is obvious that the use of intravascular imaging, either IVUS or OCT, can be beneficial not only for the optimization of stent expansion and apposition, but also for the guidance of the wire crossing, for the identification and puncture of the proximal cap and for the re-entry of the wire in ADR techniques in antegrade procedures, as well as for the identification of the two wires in CART retrograde techniques. Therefore, knowledge and skills in using intravascular imaging are essential for enhancing the possibilities for a successful and safe CTO PCI [92].

## 6. Uncrossable Lesions

The latest advances in materials and techniques for invasive revascularization of CTOs have led to the recognition of a new entity of balloon-uncrossable lesions, encountered in 5–10% of cases. These cases have high rates of technical failure, and they require specific manipulations and tricks for the completion of the procedure [93,94,95]. Before the application of special techniques in order tackle balloon-uncrossable lesions, we should apply the basic principles to increase the support of the procedure. The proper selection of the guide catheter, as well as the size of the catheter, the contact area and the angle on the reverse side of the aorta, have a primary role in optimizing the results of PCIs. The support of the guiding catheter can be increased by using a larger catheter, by increasing the active support (deep throating) or with the use of buddy wires, guide catheter extensions and side-branch anchor techniques [95].

Specialized techniques are prioritized for the optimal back up of procedures that require optimal support of the guiding catheters and start with the use of small balloons, low-profile microcatheters, plaque debulking with rotablation, subintimal modification or laser atherectomy [96]. The balloon-assisted microdissection—“BAM or grenadoplasty”—technique consists of the creation of microdissections by the high-pressure inflation and final rupture of a small compliant balloon into the proximal cap, while the Carlino technique, which is based on a forced contrast injection through a microcatheter, can lead to further dissection of the microchannels and softening of the proximal cap, allowing the crossing of the cap with a balloon [62,97]. In case of failure, the ping pong technique, in which two different catheters are used for the cannulation of the same coronary artery, providing stronger support, can be used, as well as the anchoring or trapping techniques [98,99]. The proximal anchoring distal trapping technique, which requires surfing of an antegrade wire through the collaterals to the proximal part of the retrograde vessel, can increase the support, so that the advancement of the balloons in the distal part of the CTO is feasible [95]. Another published technique for uncrossable lesions is the subintimal external crush technique [100]. According to this technique, a balloon is advanced and inflated in the subintimal space, “crushing” the CTO lesion and enabling wire and balloon crossing at the true lumen via the true lumen guidewire.

## 7. Conclusions

Although there is evidence from RCTs that PCIs of CTOs may lead to a decrease in mortality or cardiovascular events, their endorsement and utilization is continuously increasing. Real-world data from large registries with long-term follow-ups support the effectiveness of these procedures in improving survival. Moreover, recent advances in guidewires and microcatheters, as well as advances in the skills of interventional cardiologists, have increased the technical outcomes and safety of CTO procedures. Given the growth of clinical practice and the progress of modern medical technologies, it is highly likely that PCI CTOs will be advanced and used more often in patients that have previously been overlooked and treated conservatively.

## Figures and Tables

**Table 2:** The predominant wire types with their characteristics and tip loads.

Name	Vendor	Core	Tapered	Tip Load
Polymer-jacketed wires				
Fielder XT	Asahi Intecc	Stainless steel	0.009″	0.8 g
Fielder XT-A	Asahi Intecc	Stainless steel	0.010″	1 g
Fielder XT-R	Asahi Intecc	Stainless steel	0.010″	0.6 g
Fielder FC	Asahi Intecc	Stainless steel	No	0.8 g
Gladius MG	Asahi Intecc	Stainless steel	No	3 g
Gladius EX	Asahi Intecc	Stainless steel	No	3 g
Bandit	Teleflex	Stainless steel	0.009″	0.8 g
Raider	Teleflex	Stainless steel	No	4 g
Fighter	Boston Scientific	Stainless steel	0.009″	1.5 g
Pilot 50	Abbott Vascular	Stainless steel	No	1.5 g
Pilot 150	Abbott Vascular	Stainless steel	No	2.5 g
Pilot 200	Abbott Vascular	Stainless steel	No	4.1 g
Non-polymer-jacketed wires				
Gaia First	Asahi Intecc	Stainless steel	0.010″	1.7 g
Gaia Second	Asahi Intecc	Stainless steel	0.011″	3.5 g
Gaia Third	Asahi Intecc	Stainless steel	0.012″	4.5 g
Gaia Next 1	Asahi Intecc	Stainless steel	0.011″	2 g
Gaia Next 2	Asahi Intecc	Stainless steel	0.012″	4 g
Gaia Next 3	Asahi Intecc	Stainless steel	0.012″	6 g
Confianza Pro	Asahi Intecc	Stainless steel	0.009″	9 g
Confianza Pro 12	Asahi Intecc	Stainless steel	0.009″	12 g
Astato XS 20	Asahi Intecc	Stainless steel	0.008″	20 g
Astato XS 40	Asahi Intecc	Stainless steel	0.008″	40 g
MiracleBros 3	Asahi Intecc	Stainless steel	No	3 g
MiracleBros 6	Asahi Intecc	Stainless steel	No	6 g
MiracleBros 12	Asahi Intecc	Stainless steel	No	12 g
UltimateBros 3	Asahi Intecc	Stainless steel	No	0.3 g
Suoh 03	Asahi Intecc	Stainless steel	No	0.3 g
RG3	Asahi Intecc	Stainless steel	0.010″	3 g
Warrior	Teleflex	Stainless steel	0.009″	14 g
Infiltrac	Abbott Vascular	Stainless steel	0.009″	10.8 g
Infiltrac Plus	Abbott Vascular	Stainless steel	0.009″	13.9 g
Progress 40	Abbott Vascular	Stainless steel	No	5 g
Progress 80	Abbott Vascular	Stainless steel	No	11.5
Progress 120	Abbott Vascular	Stainless steel	No	17.5
Progress 140T	Abbott Vascular	Stainless steel	0.0105	15.5 g
Progress 200T	Abbott Vascular	Stainless steel	0.009″	13.5
Cross-It 100XT	Abbott Vascular	Stainless steel	0.010″	1.6 g
Cross-It 200XT	Abbott Vascular	Stainless steel	0.010″	4.4 g
Cross-It 300XT	Abbott Vascular	Stainless steel	0.010″	7.1 g
Cross-It 400XT	Abbott Vascular	Stainless steel	0.010″	10.9 g
Hornet 10	Boston Scientific	Stainless steel	0.008″	10g
Hornet 14	Boston Scientific	Stainless steel	0.008″	14 g

**Table 3:** Microcatheters used for CTO interventions.

Name	Vendor	Torqueable	Tip Profile
Braid and coil			
Corsair Pro	Asahi Intecc	Yes	1.3 Fr
Corsair Pro XS	Asahi Intecc	Yes	1.3 Fr
Turnpike	Teleflex	Yes	1.6 Fr
Turnpike LP	Teleflex	Yes	1.6 Fr
Mamba	Boston Scientific	Yes	1.4 Fr
Mamba Flex	Boston Scientific	Yes	1.4 Fr
Braided shaft			
Supercross	Teleflex	No	1.8 Fr
Caravel	Asahi Intecc	Slight rotation	1.4 Fr
Finecross M3	Terumo	Slight rotation	1.8 Fr
Finecross MG	Terumo	Slight rotation	1.8 Fr
External threaded coil			
Turnpike	Teleflex	Yes	1.6 Fr
Turnpike Spiral	Teleflex	Yes	1.6 Fr
Turnpike Gold	Teleflex	Yes	2.1 Fr
Turnpike LP	Teleflex	Yes	1.6 Fr
Dual lumen			
Sasuke	Asahi Intecc	No	1.5 Fr
Twin-Pass	Teleflex	No	2.0 Fr
Twin-Pass Torque	Teleflex	Slight rotation	2.1 Fr
Recross	IMDS	No	1.5 Fr
NHancer Rx	IMDS	No	1.5 Fr
Fineduo	Terumo	No	2.2 Fr
Steerable			
Venture	Teleflex	No	2.2 Fr
Supercross Angulated	Teleflex	No	2.4 Fr
Swiftninja	Merit	No	2.4 Fr

**Table 4:** Review of Controlled Antegrade and Retrograde Tracking (CART) techniques.

Technique	CART	r-CART	Directed r-CART	Extender r-CART
Balloon crossing	Retrograde wire	Antegrade wire	Antegrade wire	Antegrade wire
Antegrade balloon size	Based on collateral size	Large	Small	Large
Step 1	Antegrade and retrograde wire advancement and retrograde balloon delivery through collaterals at the CTO segment	Longitudinal overlap of antegrade and retrograde wires at the CTO segment	Antegrade preparation firstly with a small balloon positioned at the CTO segment	Retrograde wire advancement into the subintimal space. An antegrade wire is delivered at the planned meeting point
Step 2	Balloon inflation via the retrograde wire into the subintimal space to create an intimal/subintimal connection	Balloon inflation via the antegrade wire at the wire overlap site to create an intimal/subintimal connection	Direction of a high-torque-control retrograde wire (Asahi Gaia series) towards the antegrade inflated balloon without knuckling minimizing vessel trauma	Antegrade large balloon inflation to create an intimal/subintimal connection beyond the CTO segment
Step 3	Antegrade wire advancement through the connection to the distal true lumen	Retrograde wire advancement through the connection to the proximal true lumen	Retrograde wire advancement through the connection to the proximal true lumen	Retrograde wire advancement through the connection to the proximal true lumen

CART: Controlled Antegrade and Retrograde Tracking, CTO: chronic total occlusion, r-CART: reverse-CART.

## Abbreviations

ADR: Antegrade Dissection and Re-entry; AFR: Antegrade Fenestration and Re-entry; BASE: balloon-assisted subintimal entry; CABG: coronary artery bypass grafting; CART: Controlled Antegrade and Retrograde subintimal Tracking; CTCA: Computed Tomography Coronary Angiography; HDR: HydroDynamic contrast Recanalization; CTO: chronic total occlusion; IVUS: intravascular ultrasound; OMT: optimal medical treatment; LVEF: left ventricular ejection fraction; LVEDV: left ventricular end diastolic volume; MACE: major adverse cardiovascular event; PCI: percutaneous coronary intervention; RCT: randomized controlled trial; SAQ: Seattle angina questionnaire; RDR: Retrograde Dissection Re-entry; STAR: Subintimal Tracking and Re-entry; SWT: segmental wall thickening.
